# HPV Induces Changes in Innate Immune and Adhesion Molecule Markers in Cervical Mucosa With Potential Impact on HIV Infection

**DOI:** 10.3389/fimmu.2020.02078

**Published:** 2020-09-03

**Authors:** Alan Messala A. Britto, Livia R. Goes, Aida Sivro, Cintia Policarpo, Ângela R. Meirelles, Yara Furtado, Gutemberg Almeida, James Arthos, Claudia Cicala, Marcelo A. Soares, Elizabeth S. Machado, Ana Lúcia M. Giannini

**Affiliations:** ^1^Programa de Oncovirologia, Instituto Nacional de Câncer (INCA), Rio de Janeiro, Brazil; ^2^Laboratório de Genômica Funcional e Transdução de Sinal, Departamento de Genética, Universidade Federal do Rio de Janeiro, Rio de Janeiro, Brazil; ^3^Laboratory of Immunoregulation, National Institute of Allergy and Infectious Diseases, National Institutes of Health, Bethesda, MD, United States; ^4^Centre for the AIDS Programme of Research in South Africa (CAPRISA), Durban, South Africa; ^5^Department of Medical Microbiology, University of Manitoba, Winnipeg, MB, Canada; ^6^Department of Medical Microbiology, University of KwaZulu-Natal, Durban, South Africa; ^7^Instituto de Ginecologia, Universidade Federal do Rio de Janeiro, Rio de Janeiro, Brazil; ^8^Hospital Universitário Clementino Fraga Filho, Universidade Federal do Rio de Janeiro, Rio de Janeiro, Brazil

**Keywords:** innate immunity, toll-like receptors, interferon type I, HPV, HIV, cell junctions

## Abstract

While most HPV infections are asymptomatic and clear spontaneously, persistent infection with high-risk HPVs is associated with cervical cancer and with increased risk of HIV acquisition. Although several hypotheses have been proposed to explain this phenomenon, none has been confirmed. Our aim was to investigate the expression of host factors involved in the susceptibility to HIV infection among HPV-infected women. Cervical samples were collected to characterize the expression levels of HIV susceptibility markers in the mucosa of HPV-infected compared with HPV-uninfected women. No differences in the frequency of CCR5+, integrin α4β7+, activated and memory CD4+ T-cell were detected between the groups. We additionally evaluated the expression levels of genes involved in innate immune responses and in cell adhesion. HPV infected patients expressed higher levels of TLR9 and lower levels of pattern recognition receptors that recognize RNA (TLR3, TLR7, and MDA5/IFIH1). We also detected an impaired IFN pathway, with an increased Type I IFN and a decreased IFNα2 receptor expression. HPV+ samples displayed reduced expression of genes for adherens and tight junctions. Taken together, these results suggest that although HPV infection does not result in the recruitment/activation of susceptible CD4+ T-cell in the female genital tract, it leads to changes in the innate antiviral immune responses and in cell adhesion that are likely to favor HIV infection.

## Introduction

Human papillomavirus (HPV) is the agent of a sexually transmitted infection (STI) occurring in ~12% of the world's population (1). Most of HPV infections are asymptomatic and the host immune system is capable of clearing the virus. Nevertheless, in some cases the virus (usually high-risk HPVs—hr-HPV) subverts host defense mechanisms, causing a persistent infection that may lead to premalignant lesions and cancer (2). It has been shown that immunosuppressive diseases are associated with cervical cancer development (3), and a recent meta-analysis reinforced that HIV-infected women have a higher risk of acquiring HPV (4), potentially due to disruption of tight junctions (5, 6). The reverse scenario is also presented, and several groups showed that HPV cervical, anal or penile infections increase HIV susceptibility (7–9), suggesting that the presence of one virus is influencing the infection and pathogenesis of the other.

The chance of HIV transmission in heterosexual unprotected sex is <1% per act (10), suggesting that the female genital tract (FGT) environment acts as a key element in preventing HIV transmission due in part to physical and chemical barriers (such as mucus and epithelia) and also due to mucosal innate and adaptive immunity (11, 12). However, women continue to be disproportionally affected by HIV and STIs contribute to this effect by several mechanisms, including the recruitment of HIV susceptible cells to the infected mucosa. For instance, HSV-2 (herpes simplex virus 2), *Haemophilus ducreyi* and bacterial vaginosis (BV) cause an increase in the number of CCR5+, activated and α4β7+ integrin CD4+ T-cells in FGT (13–15), thereby enhancing HIV susceptibility (12). Higher number of Tregs, and CD4+ and CD8+ T-cell expressing α4β7 integrin are found in the cervical stroma of women with cervical intraepithelial neoplasia (CIN) lesions, but the presence, quantity and role of these cells in disease pathology is still unclear (16–19).

In addition to cellular immunity, the recognition of pathogens by pattern recognition receptors (PRRs) [such as Toll-like receptors (TLRs) and RIG-like receptors (RLRs) ] expressed by FGT cells initiate inflammatory responses through production of type I interferon (IFN-I) and other cytokines/chemokines (11), and this can both promote or inhibit viral infection/spread and activate adaptive immunity (20, 21). Moreover, HPV and inflammation induce changes in the expression of cell adhesion protein mRNAs and can alter composition and structure of keratinocyte junctions (22) and disturb epithelial barrier, thus facilitating microbial translocation (6, 23–25).

To better understand the relationship between HPV infection and HIV susceptibility, we analyzed immune responses at the FGT during HPV infection in order to identify mechanisms that could favor HIV acquisition. We assessed the frequency of HIV susceptible cells in cervical mucosa of HPV-infected women. Additionally, we evaluated the expression levels of innate immunity receptors (TLRs and RLRs), pathway mediators, cytokines, IFN-I, and IFN receptor in cervical cells from these patients, and observed changes in the innate immune responses in HPV-infected cervical tissues that may be responsible for HPV immune escape and an increased HIV susceptibility. We also evaluated mRNA expression of cell adhesion markers in the presence of HPV, observing a reduction, albeit not significant, in some of these such as E-cadherin and ZO-1, which suggest changes in FGT's capacity to function as a strong barrier against microorganisms.

## Materials and Methods

### Ethics Statement, Participant Enrolment, and Sampling

The study was approved by the Committee of Ethics in Research from the Instituto de Pediatria e Puericultura Martagão Gesteira (Rio de Janeiro, Brazil) (acceptance number: 49035215.4.0000.5264). A total of 62 women were enrolled from the Instituto de Ginecologia of Universidade Federal do Rio de Janeiro (Rio de Janeiro, Brazil) during their medical consultation in the Cervical Pathology and Colposcopy outpatient (investigating possible cervical lesions and tumors) and in the gynecology ambulatory. All of the participants provided an informed written consent. Exclusion criteria were: <18 years-old; pregnancy; menstruation at the day of the collection; abnormal cervical cytology but undetectable HPV by PCR; insufficient amount of sample; presence of chlamydia, trichomoniasis, syphilis, gonorrhea, HIV (26) or HBV (HBsAg rapid test, Bioclin, Brazil). After applying exclusion criteria, 19 HPV- (with no cervical lesions) and 24 HPV+ (with diverse grades of lesion) participants were included in the analyses. HPV diagnosis was based on PCR with My9/My11 and/or GP5+/GP6+, followed by capillary DNA sequencing and comparison of obtained sequences with related sequences from GenBank (Supplementary Figure 1, Supplementary Table 1) (26). From each patient, one cell scraper, two endocervical cytobrushes, and 5 ml of blood were collected. Samples were placed on ice, transported to the laboratory, and processed within 4 h. Cervical cells were processed as describe elsewhere (27). Briefly, cell scraper and endocervical cytobrush collections were combined and transported at 4°C in RPMI medium; cells were released from cytobrushes and cell scrapers using a 25 ml pipette and filtered in a 70 μm strainer. Half of the cells were cryopreserved or immediately used in flow cytometry; the remaining cells were frozen in RNAlater (Invitrogen, Carlsbad, CA) and used for STI diagnosis (25% of cells) and qPCR analysis (75% of cells). Total leucocytes were isolated from blood using ACK Lysing Buffer (Thermo Fisher Scientific, Waltham, MA) and were cryopreserved until further use.

### FACS Analysis

Initially, fresh cervical cells from seven HPV- and six HPV+ women were stained with CD3-FITC (UCHT-1), CCR5-PercP Cy5.5 (2A9) and CD4-APC (RPA-T4) antibodies (BD Biosciences, San Jose, CA), processed using BD Accuri C6 flow cytometer and data were analyzed using BD CSampler Software v.1.0.264.21. This equipment is set with a pre-optimized voltage and it is able to detect simultaneously epithelial cells and lymphocytes, allowing us to determine the percentage of lymphocytes among all cells collected from the cervix (Supplementary Figure 1, Supplementary Table 1). A FACS Canto II flow cytometer was also used to evaluate cervical cells and blood leucocytes. In these experiments, we evaluated further 11 HPV- and 16 HPV+ women (Supplementary Figure 1, Supplementary Table 1). For three HPV- patients, no CD4+ T-cells were detected in the cervical sample, and they were excluded from the analysis. However, their blood was used for the flow cytometry experiments. Cells were thawed and stained with CD38-FITC (HIT2), β7 integrin-PE (FIB504), CD3-PercP Cy5.5 (SP34-2), CD4-APC (RPA-T4), CD45-RA-APC-H7 (HI100), CCR5-BV-421 (2D7) antibodies (BD Biosciences) αE integrin-PE-Cy7 (B-Ly7, eBioscience) and Live/Dead Aqua (Invitrogen) and fixed with 2% paraformaldehyde. Data were analyzed using FlowJo X v.10.0.7r2. The representative gating strategies are shown in Supplementary Figure 2.

### Gene Expression Analysis

Part of the collected cervical cells stored at −80°C in RNA Later (Invitrogen) were used for mRNA extraction using MasterPure Complete DNA & RNA Purification Kit (Epicentre, Madison, WI) following manufacturer's instructions. The cDNA was synthesized from 1 μg of RNA using High Capacity cDNA Reverse Transcript Kit (Applied Biosystems, Foster City, CA).

We designed customized Taqman array plates using the following probes: GAPDH = Hs02758991_g1; RNF114 = Hs00218782_m1; RNF125 = Hs00215201_m1; UCHL1 = Hs00985157_m1 (7 HPV+/HSIL and 7 HPV- patients were assessed); TLR3 = Hs01551078_m1; TLR4 = Hs00152939_m1; TLR7 = Hs00152971_m1; TLR9 = Hs00152973_m1; DDX58 (RIG-I) = Hs00204833_m1; IFIH1 (MDA5) = Hs01070332_m1; IFNA2 = Hs00265051_s1; IFNB1 = Hs01077958_s1; IFNAR2 = Hs01022060_m1; TRIM25 = Hs01116121_m1 (11 HPV+/HSIL and 15 HPV- patients were assessed); IL-8 (CXCL8) = Hs00174103_m1; IP-10 (CXCL10) = Hs00171042_m1; MIP-1α (CCL3) = Hs00234142_m1; MIP-1β (CCL4) = Hs99999148_m1; IL-6 = Hs00174131_m1; IL-1β = Hs01555410_m1; IL-10 = Hs00961622_m1; MCP-1 (CCL2) = Hs00234140_m1; ZO-1 (zonula occludens-1) = Hs01551867_m1; Occludin = Hs01049883_m1; Claudin 1 = Hs00221623_m1; Claudin 2 = Hs01549234_m1; Claudin 4 = Hs00976831_s1; E-caderin = Hs01023895_m1 (9 HPV+/HSIL and 12 HPV- patients were assessed); TNFα = Hs00174128_m1 (4 HPV+/HSIL and 8 HPV- patients were assessed). IFNγ expression level was measured with SYBR Green using target primers: F – 5′-GGAAAGAGGAGAGTGACAGAAA-3′; R – 5′-TTGGATGCTCTGGTCATCTTTA-3′ and GAPDH primers: F – 5′-ACTGTGGATGGCCCCTCCGG-3′; R – 5′-GGGGACACGGAAGGCCATGC-3′ (10 HPV+/HSIL and 10 HPV- patients). Since we did not have enough cDNA from all patients, the number of patients used for the different analyses was variable. Reactions were performed in triplicates using a 7500 Fast Real-Time PCR System (Applied Biosystems). Target genes were normalized to the housekeeping GAPDH gene and the relative expression was calculated converting the difference in cycle thresholds (ΔCt) using the 2^−Δ*Ct*^ method.

### Heat Map

The representation of overall transcription profile was created based on a functional protein association network, the STRING database [https://string-db.org/], which includes 24,584,628 proteins from 5,090 species. STRING interaction maps are based on published direct (physical) and indirect (functional) associations. Genes were organized using the interaction graph viewer Medusa v.3.0 [https://sites.google.com/site/medusa3visualization/] (28). The image obtained is composed of nodes (target genes) and edges that indicate different kinds of interactions between the nodes. Once the network is established, we can use ViaComplex v.1.0 software [https://lief.if.ufrgs.br/pub/biosoftwares/viacomplex/] to build the heatmap. This software brings together known interaction information with expression data, yielding a functional landscape where the input genes are inserted onto the network graph taking into account their expression levels. Thus, we used the median expression data—in genes where the median HPV+ group was zero we used the mean—to calculate the “relative expression activity” (*n*_α_) of each gene. *n*_α_ assumes values between 0 and 1 and is defined as the ratio between HPV+ median expression and the sum between HPV+ and HPV- median expression. Thus, *n*_α_ <0.5 implies that expression in HPV+ group is lower than the HPV-, while *n*_α_ > 0.5 implies that HPV+ expression is higher than HPV- (29).

### Statistical Analysis

Differences in proportion of cells and in relative expression between HPV- and HPV+ women were calculated using Mann-Whitney *U*-test. Differences were considered significant when *p* ≤ 0.05. All statistical analyses and graphs were carried out using GraphPad Prism v.5.02.

## Results

### Study Participants

Clinical and demographic information of the patients enrolled in the study are shown in Supplementary Table 1. Median age of study participants was 37 (Interquatile Range (IQR) = 14). 44% of participants were HPV- and had no cytology abnormalities. In HPV+ group women had no cytotology abnormalities (*n* = 3), ASC (*n* = 3), LSIL (*n* = 3), HSIL (*n* = 12), or tumor (*n* = 1). The predominant HPV subtype was HPV16 (7/24), followed by HPV31 (6/24) and HPV66 (3/24).

### Evaluation of HIV Susceptible Cells in Cervical Mucosa

**Figure 1:**
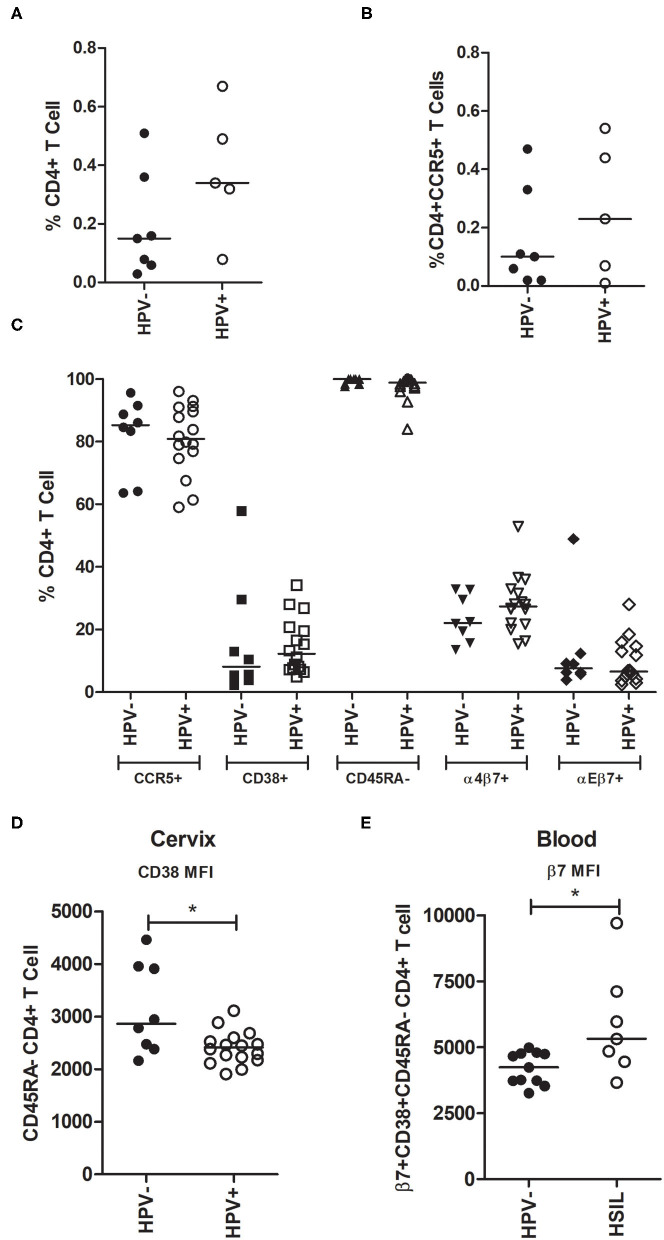
Analysis of CD4+ T-cells subsets in cervical and blood samples from HPV- and HPV+ patients. **(A,B)** Cervical cells from pap smears were stained for CD4 and CCR5 and analyzed using the BD Accuri C6 cytometer. Women were grouped into HPV- (*n* = 7) or HPV+ (*n* = 6). **(A)** Proportion of CD4+ T-cells and **(B)** CD4+CCR5+ T-cells in cervix taking into account all cells obtained from the smears (the majority are squamous and columnar epithelial cells). **(C,D)** Cervical cells were stained and analyzed using FACS Canto II. Women were grouped into HPV- (*n* = 8; no leukocytes were obtained from three of the 11 samples) and HPV+ (*n* = 16). **(C)** Frequency of CD4+ T-cells expressing either CCR5+, CD38+, CD45RA-, α4β7+, or αEβ7+ in the cervix. **(D)** CD38 MFI on memory CD45RA- CD4+ T-cells in cervical samples. **(E)** β7 MFI on memory (CD45RA-) and activated (CD38+) blood CD4+ T-cells. Blood leucocytes from HPV- (*n* = 11) and HSIL (*n* = 7) patients were analyzed using FACS Canto II. The horizontal bar represents the median value. Statistical analysis: Mann-Whitney *U*-test; *p ≤ 0.05.

Since there is evidence that HPV infection facilitates HIV acquisition, we assessed the proportion of CD4+ T-cells in the cervical samples (mainly composed of squamous and columnar epithelial cells). In this context, although the median percentage of CD4+ T-cells in all cell sample appeared higher in HPV+ samples, this difference was not statistically significant (median HPV- = 0.15% vs. HPV+ = 0.33%, *p* = 0.222, Figure 1A). We further characterized CD4+ T-cells for the presence of the CCR5 HIV co-receptor, and again no significant differences were observed (median HPV- = 0.1% vs. HPV+ = 0.23%, *p* = 0.626, Figure 1B).

To further characterize cervical CD4+ T lymphocyte subsets, in addition to CCR5, we assessed CD38, CD45RA, integrin α4β7, and integrin αEβ7 surface expression. Our results indicate that HPV infection did not significantly affect the frequency of these lymphocyte subsets in the mucosa (Figure 1C). Activation and memory markers were also analyzed in co-expression with CCR5 and integrins and no differences were observed (Supplementary Figures 3A,B). We also evaluated the median fluorescence intensity (MFI) of these receptors and found that CD38 surface expression was higher in HPV- cervical memory CD45RA-CD4+ T-cells (*p* = 0.040, Figure 1D). In addition to HPV status, patients were grouped by the presence of cervical lesions. We compared all CD4+ T-cell subsets described above between HPV- women (*n* = 8) and those with high-grade squamous intraepithelial lesion (HSIL) (*n* = 7), and we observed no significant differences (data not shown).

In addition to cervical samples, we characterized CD4+ T-cell subsets from peripheral blood and observed no significant differences between groups in cell subset frequencies (Supplementary Figures 3C–E), or in the MFI of the receptors (data not shown). However, when women were classified based on cervical lesion status, we observed that the β7 integrin MFI in CD38+CD45RA- CD4+ T-cells in the blood was higher in women who had HSIL (*p* = 0.037, Figure 1E). Altogether, these results suggest that HPV infection and cervical lesions do not have a major effect on the expression of the evaluated HIV susceptible cell subsets in the cervix, nor in the blood.

### Effect of HPV Infection on TLR and RIG Pathogen Recognition Receptors in Cervical Cells

**Figure 2:**
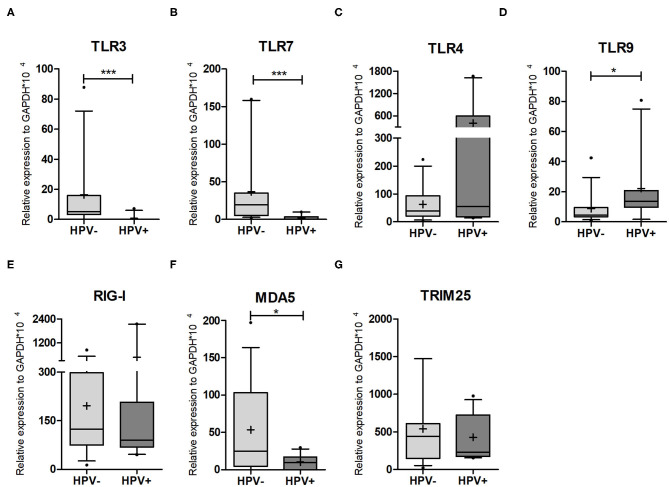
PRR expression levels in cervical cells. qPCR on HPV- and HPV+ cervical sample lysates was performed to assess the expression levels of **(A)** TLR3; **(B)** TRL7; **(C)** TLR4; **(D)** TRL9; **(E)** RIG-I/DDX58; **(F)** MDA5/IFIH1; **(G)** TRIM25. Relative expression of target genes was normalized to GAPDH (2^−Δ*Ct*^). Box plots represent 10–90 percentile; dots represent outlier samples; “+” represents the mean and the horizontal bar, the median. HPV- = 15 and HPV+ = 11 women. Mann-Whitney *U*-test; *p ≤ 0.05; ***p ≤ 0.001.

Next, we decided to evaluate the expression levels of genes involved in pathogen recognition and innate immunity of cervical cells by qPCR, in order to assess if any differences detected could account for the higher HIV susceptibility in HPV-infected women. In this context, TLR3, which recognizes dsRNA, was not expressed in nine out of 11 HPV+ women (81.8%), while only two healthy controls did not express this receptor (13%) (Figure 2A). TLR7 (triggered by ssRNA) was not expressed in eight out of 11 HPV+ patients (72.7%) (Figure 2B). On average, HPV- samples expressed more than 17 times these two endossomal receptors compared to HPV+ counterparts, suggesting that HPV infection reduces or abrogates their expression (Supplementary Table 2). TLR4 is expressed at the plasma membrane and recognizes not only bacterial LPS, but also the HIV envelope and the HPV capsid protein (5, 30). The expression levels of this receptor were not affected by HPV infection (median: HPV- = 38.4; HPV+ = 55) (Figure 2C, Supplementary Table 2). We also assessed TLR9, which recognizes dsDNA (including the HPV genome). The expression of this receptor was 2.5-fold higher in HPV-infected mucosa (Figure 2D, Supplementary Table 2). Taken together, these data indicate that HPV infection affects the expression of TLRs involved in recognition of nucleic acids, increasing the levels of DNA recognition receptor, and decreasing the expression of TLRs that recognize RNAs.

We next investigated the expression levels of the cytoplasmic receptors RIG-I/DDX58 and MDA5/IFIH1. RIG-I/DDX58 expression levels were not affected by the presence of HPV, while MDA5/IFIH1 was significantly downregulated by HPV infection (Figures 2E,F, Supplementary Table 2). Concerning RLR pathway regulators, we assessed the expression of ubiquitin ligase TRIM25, a positive regulator of RIG-I/DDX58, but detected no significant difference between the groups (*p* = 0.9596) (Figure 2G, Supplementary Table 2). We also assessed the levels of the ubiquitin ligase RNF114, which has a controversial role in RLR response, as well as the RLR negative regulator RNF125. The expression levels of both genes were similar, independently of HPV status (Supplementary Figures 4A,B, Supplementary Table 2). Finally, we assessed the levels of UCHL1 (a deubiquitinating enzyme that attenuates NFκB signaling) whose expression was shown to be increased in HPV16-infected primary keratinocytes (31). However, no differences were detected in our samples (Supplementary Figure 4C). Altogether, the expression data from TLRs and RLRs corroborate the idea that HPV infection impairs the recognition of exogenous RNAs.

### Effect of HPV Infection on Cytokine mRNA Expression

**Figure 3:**
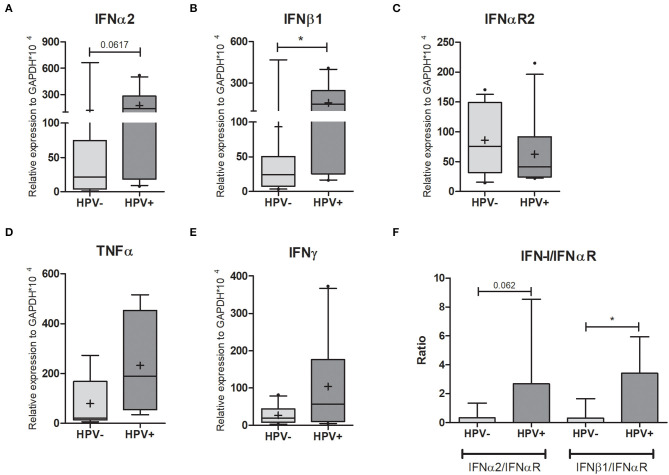
Cytokine expression in cervical cells. Expression levels of **(A)** IFNα2; **(B)** IFNβ1; **(C)** IFNαR2; **(D)** TNFα, and **(E)** IFNγ relative to GAPDH expression. Box plots represent 10–90 percentiles; dots represent outlier sample; “+” represents the mean and the horizontal bar, the median. In **(F)** the IFN-I/IFNαR2 ratios are presented in a histogram depicting medians and with interquartile range. HPV- = 15 and HPV+ = 11 women **(A,B,C,F)**; HPV- = 8 and HPV+ = 4 women **(D)**; HPV- = 10 and HPV+ = 10 women **(E)**. Mann-Whitney *U*-test; *p ≤ 0.05.

HPV is known to modulate cytokine production, impairing pro-inflammatory response, possibly as a strategy to evade the host immune system, ultimately resulting in development of lesions in patients in which this modulation is successful (2). Focusing on Type I IFN antiviral responses we quantified the expression levels of IFNα2, IFNβ1, and IFNαR2 (IFN-I receptor) in cervical samples by qPCR. Both type I IFNs were at least five times more expressed in HPV+ women (Figures 3A,B, Supplementary Table 2), yet the expression of IFN receptor (IFNαR2) halved (Figure 3C, Supplementary Table 2). By calculating the IFNα2/IFNαR2 and IFNβ1/IFNαR2 ratios, we observed that they were 9- and 11-fold higher in HPV+ women compared with controls, respectively (Figure 3F). We also assessed the expression levels of IFNγ and TNFα, and in both cases no significant differences were detected, despite a 3-fold and 9-fold increase in HPV+ group, respectively (Figures 3D,E, Supplementary Table 2). We then measured the expression levels of MCP-1, MIP-1α, MIP-1β, IL-8, IP-10, IL-6, IL-1β, and IL-10, cytokines previously associated to HPV infection and HIV susceptibility (9, 20) and no differences in expression were detected between groups (Supplementary Figures 4D–I, Supplementary Table 2). These data suggest that although the infected keratinocytes are producing higher levels of type I IFNs, their antiviral response could be impaired due to decreased IFN receptor expression in the same cells. Hence, the infected cells are not able to initiate a robust response potentially facilitating HPV persistence. This could also partially explain the lack of observable differences in cytokine mRNA expression levels between the two groups.

### HPV Reduces Adhesion Molecule Expression

**Figure 4:**
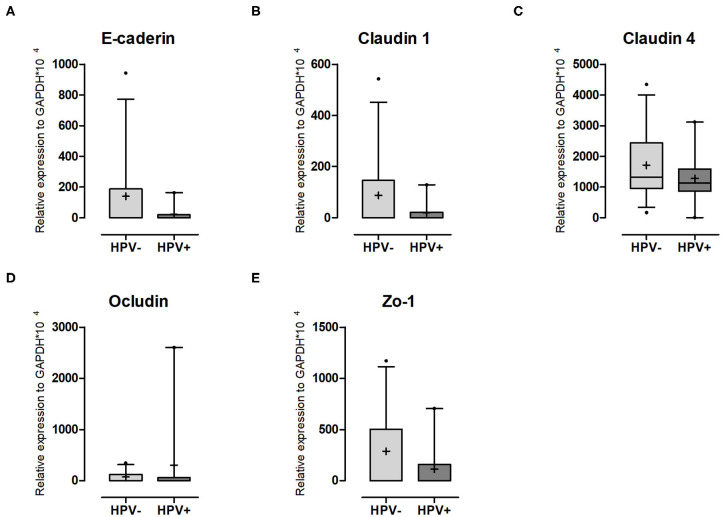
Adhesion molecule expression in cervical cells. Expression levels of **(A)** E-caderin; **(B)** Claudin-1; **(C)** Claudin-4; **(D)** Ocludin and **(E)** ZO-1 relative to GAPDH expression. Box plots represent 10–90 percentiles; dots represent outlier samples; “+” represents the mean and the horizontal bar, the median. HPV- = 12 and HPV+ = 9 women. Statistical test: Mann-Whitney *U*-test.

It has been described in the literature that inflammation leads to increased tissue permeability (23, 32, 33). The increase observed in the mRNA levels of both TNFα and IFNγ led us to think that it could result in a weakened epithelial barrier. Thus, we decided to evaluate this possibility by quantifying gene expression for cell-cell contact markers. Although the differences were not significant, for the adherens junction marker E-cadherin a 6-fold decrease was seen (Figure 4A, Supplementary Table 2). For the tight junction markers Claudin 1, Claudin 4, and ZO-1, reductions were in the order of 4.5, 1.4, and 2.5-fold, respectively (Figures 4B,C,E, Supplementary Table 2). No patient expressed Claudin 2 (Supplementary Table 2) and only Occludin was higher in the HPV+ group (Figure 4D, Supplementary Table 2). If the effects we see are directly or indirectly mediated by the presence of the virus is not addressed here and will be investigated in future studies.

**Figure 5:**
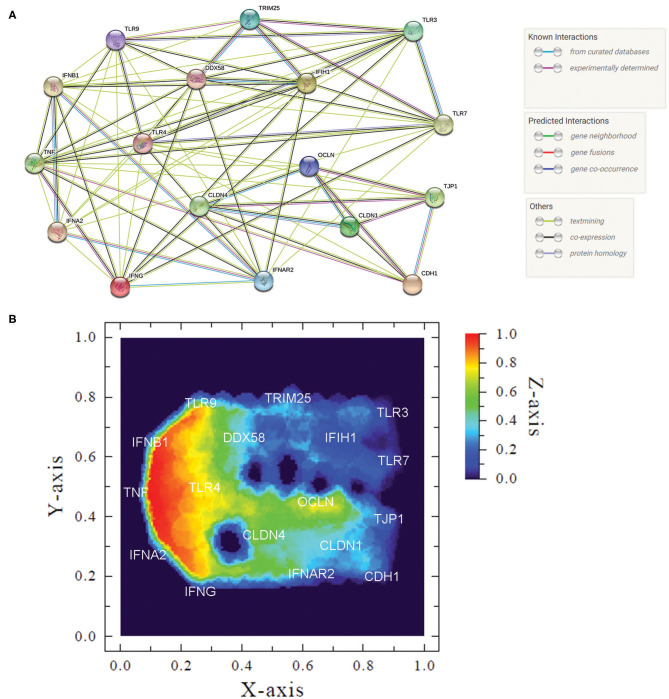
Measured innate immune and adhesion gene expression patterns and gene interactions. **(A)** Protein network of analyzed genes showing action types and effects of proteins (created using string-db.org). **(B)** Heatmap of gene expression patterns comparing HPV- women with HPV+ counterparts. We used median to calculate the “relative expression activity” (*n*_α_), except for TLR3, TLR7, E-caderin, Claudin-1, Occludin, and ZO-1, in that the HPV+ group was zero and we used the mean. In this graph, coordinates (X- and Y-axis) represent normalized values of the input network topology. Color gradient (Z-axis) represents the *n*_α_ value in the color scale. Red signal represents high expression and blue, low expression in HPV+ compared with HPV- group.

To summarize our findings, we created an interaction map depicting the targets evaluated in this work (Figure 5A) and built a heatmap representing the transcription profile of the analyzed genes comparing HPV- with HPV+ cervical cells (Figure 5B). Although this map is based not only on confirmed but also on putative interactions based on *in silico* information, it helps us to visualize the interconnections among all of these targets. In the heatmap, built with expression levels of these genes, we can observe clusters that are differentially expressed in HPV-infected mucosa. A cluster of upregulated genes in HPV+ women contained IFNs-I, IFNγ, TNFα, and TLR9, while genes involved in RNA recognition (RLR pathway, TLR3, and TLR7) and adhesion molecules formed a cluster of downregulated transcripts (Figure 5B). Taken together, the data suggest that HPV infection promotes a controlled inflammatory response able to reduce or abrogate the expression of innate immunity receptors triggered by RNA viruses. Moreover, an HPV role in changing epithelial integrity either directly or indirectly might render the female genital tract more susceptible to HIV infection.

## Discussion

A number of studies have documented an association between HPV infection and increased risk of HIV acquisition (7, 9, 34–38), however the immune mechanisms that mediate this effect remain largely unexplored. In this study we investigated the underlying mechanism by which HPV increases HIV susceptibility. To this end we analyzed samples from both HPV-infected and uninfected women from the Ginecology clinic of Universidade Federal do Rio de Janeiro. The analysis focused on the cellular and transcription immune responses from FGT and blood. Our data indicated that the HPV status does not have a profound effect on cellular activation and HIV susceptibility markers in cervical and systemic compartments. However, we observed a significantly decreased mRNA expression of PRRs that recognize RNA (including TLR3/7 and MDA5) in cervical samples from HPV infected women. While we observed a significant increase in the expression of Type I IFNs in HPV infected samples, we also observed a decrease in IFNαR2 indicative of impaired antiviral signaling pathway. A decreased expression of cell adhesion genes was also detected in HPV+ samples.

As expected, the vast majority of cells in cervical samples are epithelial and CD4+ T-cells account for <1% of the total cell count. Although we detected slightly more CD4+ T-cells in HPV-infected samples, this was not significant. When we assessed the expression of HIV susceptibility markers (including CCR5, α4β7, αEβ7, and CD38) on total and memory CD4+ T-cells from the cervix, no differences were detected between HPV infected and uninfected patients, similar to previous studies (39). However, we did observe a significant reduction in CD38 MFI on memory CD4+ T-cells from HPV infected samples. Previous studies have reported conflicting results with regard to CD4+ T-cell numbers and profiles in HPV infection. While some authors describe an increased number of CD4+ T lymphocytes both in epithelium and stroma of CIN lesions compared to normal cervix (17, 19), others found no differences (16). Our results suggest that increased HIV susceptibility in HPV-infected women is likely not the consequence of increased CD4+ T-cell recruitment to the cervix or increased target cells activation.

When we examined the blood samples, as expected, we observed no major differences in CD4+ T-cell phenotypes between HPV infected and uninfected samples. However, patients with HSIL had significantly higher expression of β7 on systemic, activated, memory CD4+ T-cells compared to HPV uninfected samples. Due to the nature of HPV infection we do not expect it to have a significant effect on systemic immune response (40); however localized mucosal inflammation could still potentially affect the systemic expression of lymphocyte trafficking markers, such as integrin α4β7. Increased expression of α4β7 on memory CD4+ T-cells in the blood was previously associated with high risk of HIV acquisition and disease progression (41), and this could potentially contribute to the higher HIV susceptibility in HPV infected women.

We also evaluated the expression levels of PRRs that play a key role in innate immune response. For TLR3 and TLR7, that recognize dsRNA and ssRNA, respectively, both were downregulated or not expressed in HPV-infected women. Our results are corroborated by a case-control study comparing cervical biopsies from healthy and carcinoma samples, where a reduction of TLR3 expression—both at mRNA and protein levels—was found in the presence of HPV (42). However, studies that analyzed cytobrush samples of CIN detected no TLR3 differences even in patients with persistent infection (43–45), and others showed increased expression of this TLR in carcinoma and in dysplastic epithelium (43, 46, 47). Regarding TLR7, different approaches showed maintenance (42, 44, 47) or increase (43, 46, 48) of its expression levels in the context of HPV infection. To our knowledge, our study is the first to show TLR7 reduction in HPV-positive samples. We detected an increased expression of TLR9 in HPV-infected mucosa, as reported previously (44, 47). With respect to TLR4 expression in the cervix, data in the literature are controversial, with some groups detecting an increase (45, 46) and others a reduction (42) during HPV infection or cancer development. Herein, no differences in TLR4 expression were found between HPV- and HPV+ cervical samples. This has been shown by others comparing healthy mucosa with CIN and cervical cancer (43, 44, 47). Overall, we demonstrate a significantly decreased expression of TLR3 and 7 and significantly increased expression of TLR9 in cervical samples from HPV infected women. The observed changes in pathogen recognition receptors and downstream innate immune signaling in hr-HPV could contribute to changes in FGT cytokine/ chemokine profiles recently linked to HPV- mediated increase in HIV acquisition (9).

RIG-I/DDX58 and MDA5/IFIH1 are cytoplasmic receptors that recognize RNA of different structures and lengths (49). There is limited work done with RLRs in keratinocytes and the majority of the previous studies analyzed the expression levels after Poli(I:C) stimulation in cells infected or not by HPV (50–52). We observed a reduction in MDA-5/IFIH1 expression, but RIG-I/DDX58 levels were not altered. Our results are partially corroborated by others (52), who detected a decrease both of MDA-5/IFIH1 and RIG-I/DDX58 in hr-HPV+ keratinocytes. Since TLR3 and MDA5/IFIH1 are in part responsible for NF-κB and IRF3 activation, respectively (50), HPV infection seems to partially impair both pathways.

When we looked at the antiviral Type I IFNs, IFNα2, and IFNβ1, their expression was significantly upregulated in hr-HPV infected samples. These results were not expected since it is known that HPV reduces IFN-I production mainly via viral proteins E6 and E7 (2, 50, 53). We think our data can be partly explained by the higher levels of TLR9 in HPV+ cervix, since it has been suggested that TLR9 supports a sustained inflammatory response in persistent HPV infection that is not sufficient for clearance but enough to activate cervical inflammation, inducing cancer progression (44). We confirmed previous data which used RT-PCR to detect the presence of IFNαR-1 and IFNαR-2 transcripts in control or HPV+ biopsies. The findings indicated reduced or no expression of both in HPV+ samples (54). Using RT-qPCR, we indeed measured IFNαR2 expression and saw an 80% decrease compared to HPV- controls. These data suggest that HPV infection regulates not only the IFN signaling cascade components such as Tyk2, STAT1, and IRF9 (55), but also the IFN-I receptor itself, indicating the existence of yet another mechanism for HPV evasion of the immune system. Since IFNβ1 has been shown to downregulate IFNαR2 cell surface expression (56), we can envisage a scenario whereby the HPV-infected keratinocytes are producing more type I IFNs in the mucosa and that these cytokines might be inhibiting IFN receptor cell surface expression in surrounding immune cells, leading to decreased interferon stimulated gene induction and reduced antiviral response. This could be an important contributing factor, not only to HPV persistence and cervical cancer development, but also to increased susceptibility to HIV considering that reduced activity of type I IFN receptor delay/decrease immune responses and accelerate AIDS progression and that high levels of type I IFN at first induces antiviral response protecting mucosa, but increase HIV susceptibility repressing several IFN effects in sustained high level condition (57).

In addition to type I IFN, we analyzed the expression levels of cytokines previously associated to HIV susceptibility and/or differentially expressed upon HPV infection (MCP-1, MIP-1α, MIP-1β, IL-8, IP-10, IL-6, IL-1β, IL-10, IFNγ, and TNF-α) (9, 20, 58, 59), but we did not find significant differences between HPV- and HPV+ women. Despite the low number of participants, our data match the ones obtained in studies where HPV infection had little or no effect on cytokine production (2, 39, 59, 60). Although IFNγ and TNFα were not statistically different between the two groups, they were at least three times higher in the HPV+ group. These cytokines and type I IFNs are able to impair epithelial cell permeability (23, 61–63), hence we measured expression levels of E-caderin, claudins 1, 2, and 4, occludin 1, and ZO-1, adhesion markers that support epithelial tissue integrity, and observed reduction for majority of them. The literature reinforces that HPV infection and induced lesions reduce E-caderin expression (64, 65) and affect tight junctions (66, 67). This reduction might perturb the strength of cell-cell, contacts enabling HIV access to immune cells embedded in the cervical mucosa tissue. Future experiments performing immune staining to confirm whether mRNA results reflect protein levels should be done to confirm this hypothesis.

Our study has several limitations including a small number of patients, the lack of histological staining, cytokine protein measurements and *in vitro* HIV infection assay using patients mucosal cells. Despite these, we analyzed four aspects of HPV infection which could render the host more susceptible to HIV: (1) presence of HIV target cells in the HPV-infected mucosa; (2) expression of cytokines; (3) expression of innate immunity genes; (4) expression of cell adhesion molecules by mucosal cells. As far as we are aware, this is the first study that aimed at these four aspects simultaneously in this population. Our data indicate no differences in amounts or phenotypes of T-cells in HPV-infected mucosa, but characterizes the effect of hr-HPV infection on mucosal immune responses and permeability. Furthermore, the joint analysis of gene interaction and expression revealed clusters of HPV-upregulated—antiviral and inflammatory cytokines—and downregulated genes—PRRs that recognize RNA, IFNαR2 and adhesion proteins—suggesting that HPV either directly or indirectly modulates genes with similar functions. Altogether, mucosal inflammation, reduced ability to respond to immune stimulation and decreased expression of cell adhesion markers could facilitate HIV establishment in the female genital tract. Considering that the effects we observed are specific to hr-HPV infection, this further highlights the need for better HPV vaccination coverage, especially in the areas of high HIV incidence.

## Data Availability Statement

All datasets generated for this study are included in the article/Supplementary Material.

## Ethics Statement

The studies involving human participants were reviewed and approved by Comitê de Ética em Pesquisa do Instituto de Puericultura e Pediatria Martagão Gesteira da Universidade Federal do Rio de Janeiro (CEP/IPPMG). The patients/participants provided their written informed consent to participate in this study.

## Author Contributions

AB, AG, and EM: conceptualization and writing original draft. AB, CP, ÂM, YF, and GA: sample collection. AB, LG, AS, AG, and EM: flow cytometer experiment and analysis. AB, CP, AG, and EM: gene expression experiment and analysis. JA, CC, MS, AG, and EM: funding acquisition. AB, AG, EM, CC, LG, AS, and MS: writing, reviewing, and editing. All authors contributed to the article and approved the submitted version.

## Conflict of Interest

The authors declare that the research was conducted in the absence of any commercial or financial relationships that could be construed as a potential conflict of interest.
