# Supplementary material for: HPV Induces Changes in Innate Immune and Adhesion Molecule Markers in Cervical Mucosa With Potential Impact on HIV Infection
Source: Front Immunol. 2020 Sep 3;11:2078. doi: 10.3389/fimmu.2020.02078 (PMC7494736; doi:10.3389/fimmu.2020.02078)
Supplement: Supplementary file 1 [file Image_1.pdf]

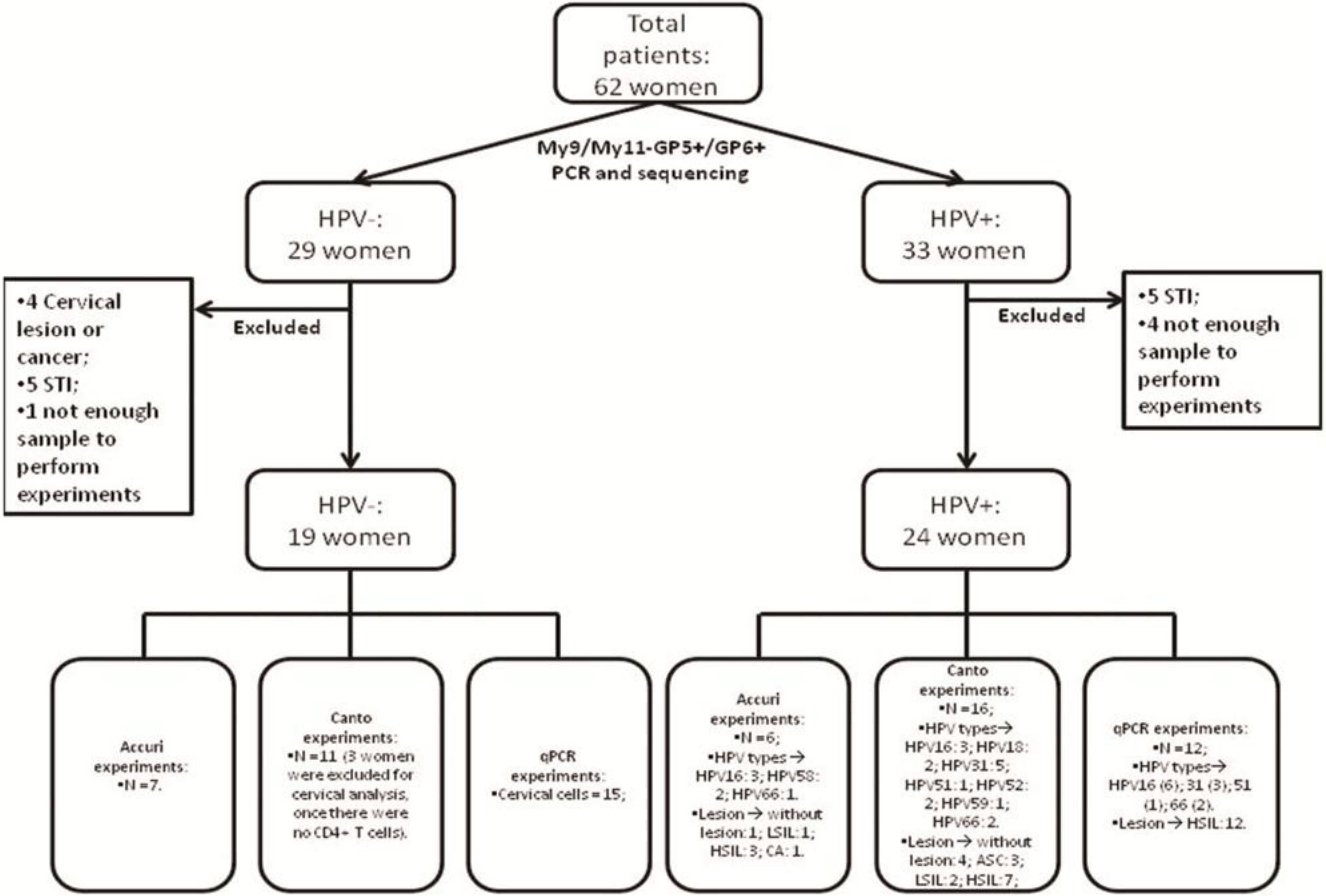
Total patients: 62 women
My9/My11-GP5+/GP6+ PCR and sequencing
HPV-: 29 women
HPV+: 33 women
Excluded
•4 Cervical lesion or cancer; •5 STI; •1 not enough sample to perform experiments
Excluded
•5 STI; •4 not enough sample to perform experiments
HPV-: 19 women
HPV+: 24 women
Accuri experiments: •N =7.
Canto experiments: •N =11 (3 women were excluded for cervical analysis, once there were no CD4+ T cells).
qPCR experiments: •Cervical cells = 15;
Accuri experiments: •N =6; •HPV types→ HPV16: 3; HPV58: 2; HPV66: 1. •Lesion→ without lesion: 1; LSIL: 1; HSIL: 3; CA: 1.
Canto experiments: •N =16; •HPV types→ HPV16: 3; HPV18: 2; HPV31: 5; HPV51: 1; HPV52: 2; HPV59: 1; HPV66: 2. •Lesion→ without lesion: 4; ASC: 3; LSIL: 2; HSIL: 7;
qPCR experiments: •N =12; •HPV types→ HPV16 (6); 31 (3); 51 (1); 66 (2). •Lesion→ HSIL: 12.

Supplementary Figure 1. Flow diagram of patients' selection criteria. STI: sexually transmitted infection; HPV types: classification based on sequencing of My9/My11 and GP5+/GP6+ PCR product; Lesion: based on cytology; ASC – atypical squamous cells; LSIL – low grade squamous intraepithelial lesion; HSIL – high grade squamous intraepithelial lesion; CA – cervical cancer; Accuri experiments: patients information of experiments that were carried out using BD Accuri C6 as flow cytometer; Canto experiments: patients information of experiments that were carried out using FACSCanto II as flow cytometer; qPCR experiments: patients information of qPCR experiments.
