# Supplementary material for: HPV Induces Changes in Innate Immune and Adhesion Molecule Markers in Cervical Mucosa With Potential Impact on HIV Infection
Source: Front Immunol. 2020 Sep 3;11:2078. doi: 10.3389/fimmu.2020.02078 (PMC7494736; doi:10.3389/fimmu.2020.02078)
Supplement: Supplementary file 2 [file Image_2.pdf]

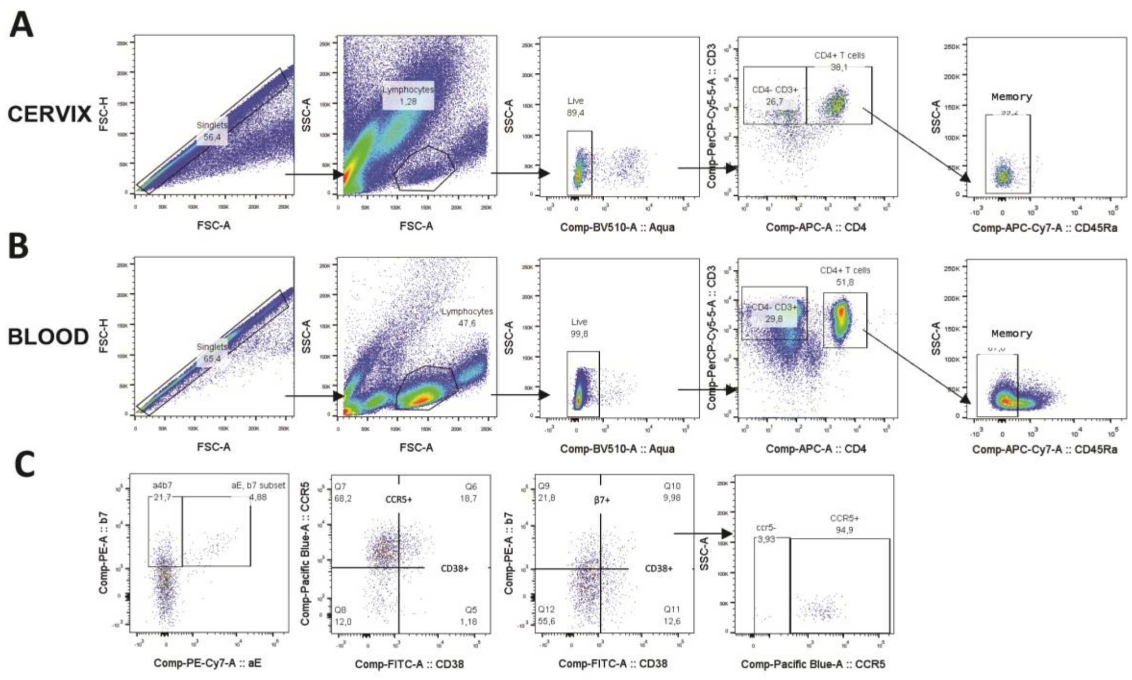

Supplementary Figure 2. Gating strategy. Representative gating strategy in **(A)** cervix and **(B)** blood for CD4+ T cells (second to last panel) and CD45RA- CD4+ T cells (last panel). Cells were gated on singlets, lymphocytes, live cells, CD3+CD4+ and CD45RA- cells. **(C)** Representative gating strategy for CD4+ T cells and CD45RA- CD4+ T cells expressing and coexpressing β7, αE, CCR5 and CD38. α4β7+ - cells β7+ and αE-; αEβ7+ - cells cells β7+ and αE+.
