# Supplementary material for: HPV Induces Changes in Innate Immune and Adhesion Molecule Markers in Cervical Mucosa With Potential Impact on HIV Infection
Source: Front Immunol. 2020 Sep 3;11:2078. doi: 10.3389/fimmu.2020.02078 (PMC7494736; doi:10.3389/fimmu.2020.02078)
Supplement: Supplementary file 3 [file Image_3.pdf]

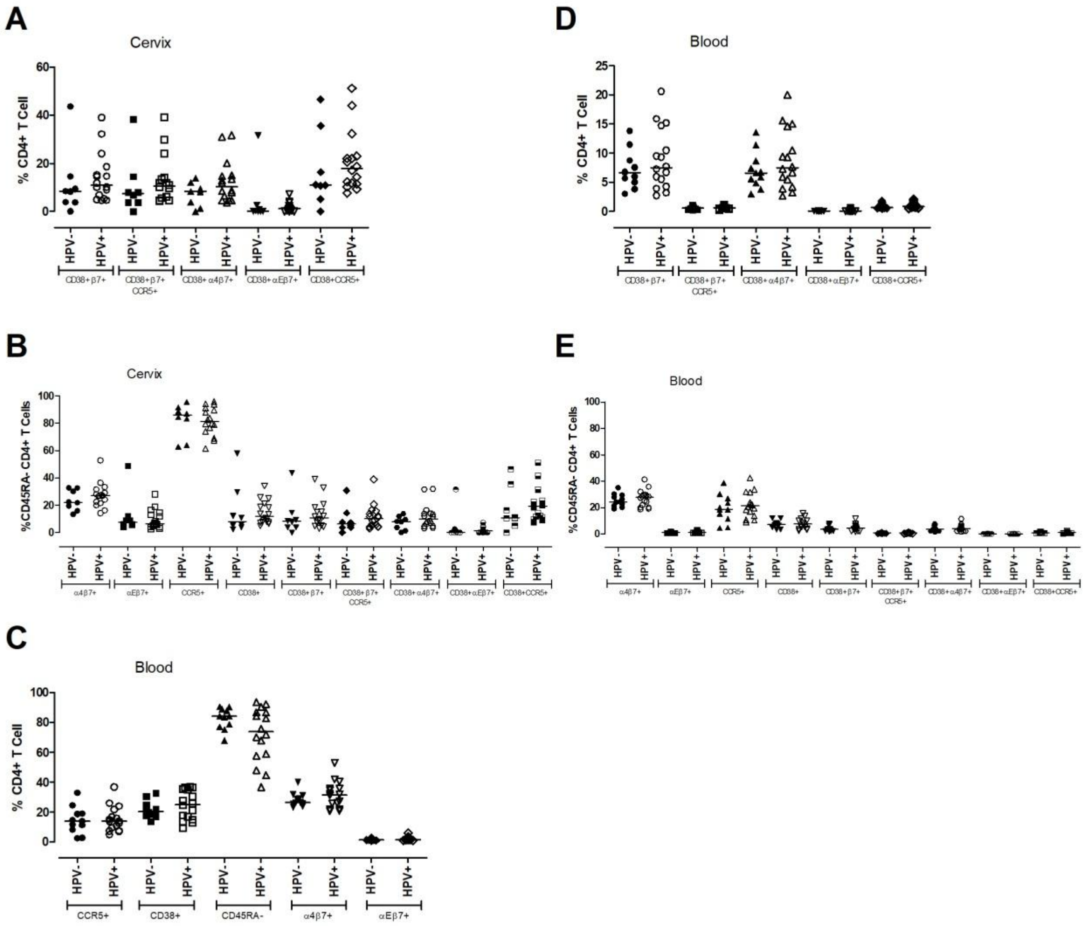

Supplementary Figure 3. **Analysis of CD4+ T-cells and memory CD45-CD4+ T-cells subsets in patients' cervix and blood.** Percentage of total CD4+ T cells **(A)** and memory CD45RA-CD4+ T cells **(B)** co-expressing several combinations of CD38, CCR5, β7 and αE integrin in cervical mucosa from HPV- (n = 8) or HPV+ (n = 16) samples. Percentage of CD4+ T-cells expressing CCR5+, CD38+, CD45RA-, α4β7+ or αEβ7+ **(C)** and of total CD4+ T cells **(D)** and memory CD45RA-CD4+ T cells **(E)** co-expressing several combinations of CD38, CCR5, β7 and αE integrin in blood from HPV- (n = 11) or HPV+ (n = 16) samples. Data were collected using a FACS Canto II apparatus. The horizontal bar in each set represents the median value. Mann-Whitney *U* test was used to assess.
