# Supplementary material for: HPV Induces Changes in Innate Immune and Adhesion Molecule Markers in Cervical Mucosa With Potential Impact on HIV Infection
Source: Front Immunol. 2020 Sep 3;11:2078. doi: 10.3389/fimmu.2020.02078 (PMC7494736; doi:10.3389/fimmu.2020.02078)
Supplement: Supplementary file 4 [file Image_4.pdf]

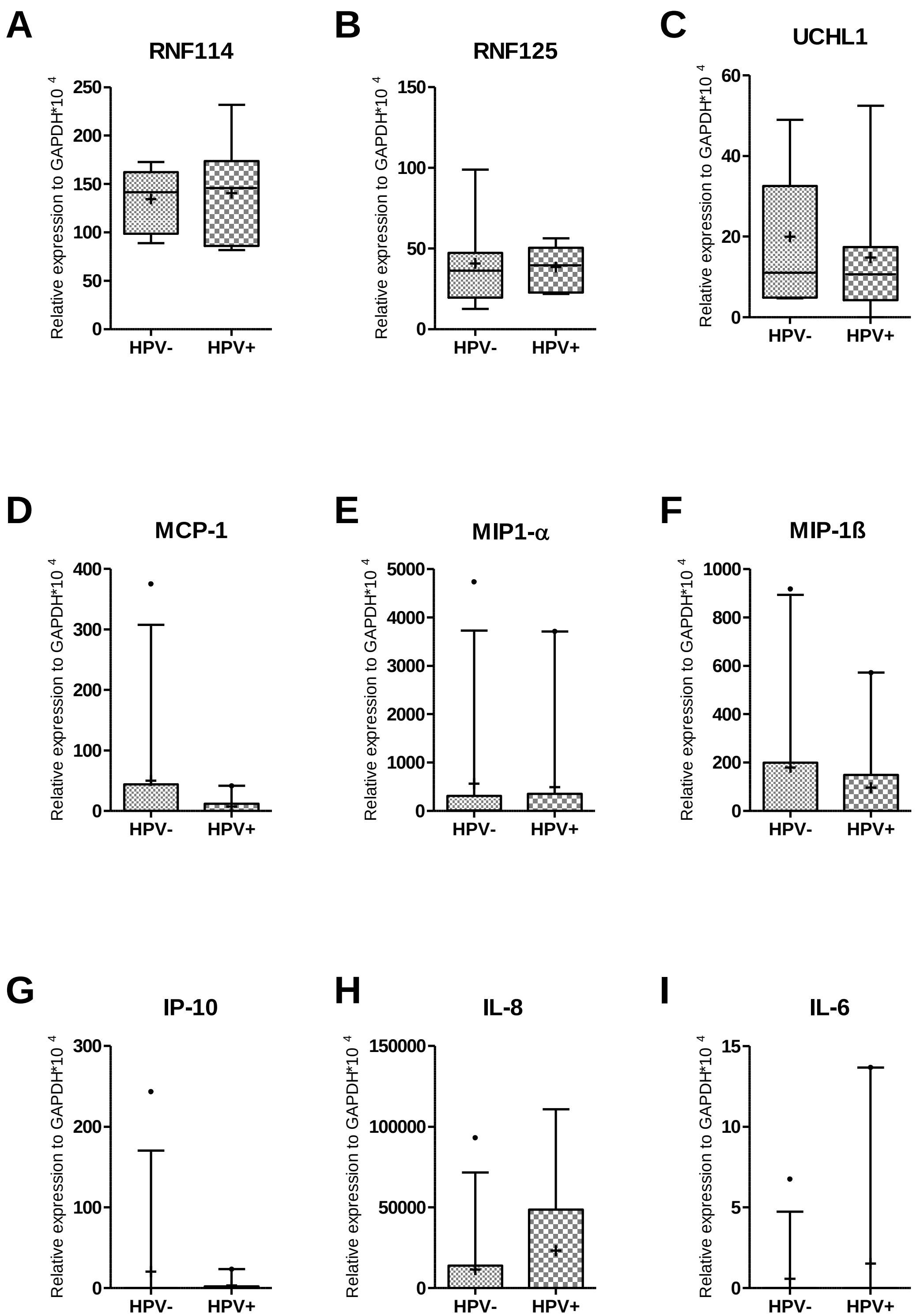

Supplementary Figure 4. **Expression levels of PRR regulators and cytokines.** qPCR on HPV- and HPV+ cervical samples were performed to assess expression levels of: **(A)** *RNF114*; **(B)** *RNF125*; **(C)** *UCHL1*; **(D)** *MCP-1*; **(E)** M*IP-1α*; **(F)** *MIP-1β (*; **(G)** *IP-10*; **(H)** *IL-8* and **(I)** *IL-6*. Relative expression of target genes was normalized to *GAPDH* ($2^{-\Delta Ct}$). Boxes represent 10-90 percentiles; dots represent outliers; "+" is the mean and the horizontal bar is the median value. HPV- = 7 and HPV+ = 7 women (A, B, C); HPV- = 12 and HPV+ = 9 women (D, E, F, G, H, I). Mann-Whitney *U* test was used for statistical analysis.
