# Supplementary material for: HPV Induces Changes in Innate Immune and Adhesion Molecule Markers in Cervical Mucosa With Potential Impact on HIV Infection
Source: Front Immunol. 2020 Sep 3;11:2078. doi: 10.3389/fimmu.2020.02078 (PMC7494736; doi:10.3389/fimmu.2020.02078)
Supplement: Supplementary file 5 [file Table_1.docx]

**Supplementary Table 1**. Clinical and demographic patient information.

| **ID** | **HPV type** | **Cytology abnormalities** | **Age** | **Flow cytometry** | **qPCR** |
| --- | --- | --- | --- | --- | --- |
| **IG01** | - | None | 38 | Accuri | - |
| **IG07** | - | None | 42 | Accuri | Yes |
| **IG09** | - | None | 61 | Accuri | Yes |
| **IG10** | - | None | 44 | Accuri | - |
| **IG12** | - | None | 23 | Accuri | - |
| **IG14** | - | None | 45 | Accuri | Yes |
| **IG20** | - | None | 37 | Accuri | Yes |
| **IG25** | - | None | 62 | - | Yes |
| **IG29** | - | None | 61 | Canto | - |
| **IG34** | - | None | 59 | Canto | Yes |
| **IG41** | - | None | 33 | Canto | Yes |
| **IG42** | - | None | 35 | Canto | Yes |
| **IG44** | - | None | 65 | Canto | Yes |
| **IG46** | - | None | 39 | Canto | Yes |
| **IG47** | - | None | 57 | Canto | Yes |
| **IG51** | - | None | 43 | Canto | Yes |
| **IG56** | - | None | 30 | Canto | Yes |
| **IG58** | - | None | 33 | Canto | Yes |
| **IG59** | - | None | 40 | Canto | Yes |
| **IG03** | 16 | HSIL | 40 | Accuri | Yes |
| **IG13** | 66 | HSIL | 31 | Accuri | Yes |
| **IG15** | 16 | Tumor | 43 | Accuri | - |
| **IG17** | 58 | LSIL | 35 | Accuri | - |
| **IG18** | 58 | None | 29 | Accuri | - |
| **IG19** | 16 | HSIL | 39 | Accuri | Yes |
| **IG21** | 31 | HSIL | 31 | Canto | Yes |
| **IG22** | 16 | HSIL | 42 | Canto | Yes |
| **IG26** | 31 | None | 36 | Canto | - |
| **IG28** | 16 | HSIL | 31 | Canto | Yes |
| **IG30** | 31 | LSIL | 29 | Canto | - |
| **IG31** | 51 | HSIL | 34 | Canto | Yes |
| **IG32** | 16 | HSIL | 24 | Canto | Yes |
| **IG33** | 31 | HSIL | 32 | Canto | Yes |
| **IG36** | 66 | HSIL | 59 | Canto | Yes |
| **IG37** | 59 | ASC | 35 | Canto | - |
| **IG40** | 31 | None | 29 | Canto | - |
| **IG45** | 52 | LSIL | 35 | Canto | - |
| **IG48** | 18 | None | 37 | Canto | - |
| **IG49** | 18 | None | 49 | Canto | - |
| **IG52** | 52 | ASC | 29 | Canto | - |
| **IG55** | 66 | ASC | 65 | Canto | - |
| **IG62** | 31 | HSIL | 53 | - | Yes |
| **IG63** | 16 | HSIL | 28 | - | Yes |

ID – patients identification code; Accuri – Cervical cells and leucocytes were used in experiments with BD Accuri Flow Cytometer; Canto - Cervical cells and leucocytes were used in experiments with FACSCanto II Flow Cytometer; yes – samples used for qPCR assays.
