# Supplementary material for: HPV Induces Changes in Innate Immune and Adhesion Molecule Markers in Cervical Mucosa With Potential Impact on HIV Infection
Source: Front Immunol. 2020 Sep 3;11:2078. doi: 10.3389/fimmu.2020.02078 (PMC7494736; doi:10.3389/fimmu.2020.02078)
Supplement: Supplementary file 6 [file Table_2.docx]

**Supplementary Table 2**. Median and mean relative expression of target genes to GAPDH x10^4^ among HPV- and HPV+ women.

| **Gene** | **HPV-** | | **HPV+11)** | | ***p*-value** |
| --- | --- | --- | --- | --- | --- |
|  | **Median(IQR)** | **Mean(SD)** | **Median(IQR)** | **Mean(SD)** |  |
| ***TLR3* ^a^** | 5.3(12.5) | 16.5(±25.1) | 0(0) | 0.8(±2.2) | 0.0008* |
| ***TLR4* ^a^** | 38.4(73.2) | 63.1(±64.5) | 55(574.9) | 401.4(±610.4) | 0.3521 |
| ***TLR7* ^a^** | 19.6(29.7) | 36.8(±51.2) | 0(3.6) | 2(±3.9) | 0.0004* |
| ***TLR9* ^a^** | 4.5(6.2) | 8.9(±10.7) | 13.5(11.3) | 22.1(±25.5) | 0.0195* |
| ***RIG-I/DDX58* ^a^** | 124.4(222.4) | 195.8(±202.8) | 90.2(138) | 474.7(±821.5) | 0.8355 |
| ***MDA5/IFIH1* ^a^** | 24.8(192.6) | 53.7(±61.2) | 9.4(17.3) | 10.4(±10.1) | 0.0472* |
| ***TRIM25* ^a^** | 440.6(464) | 540.2(±542) | 231.4(549.3) | 427.5(±297.8) | 0.9586 |
| ***IFNα2* ^a^** | 21.4(70.9) | 121(±277.5) | 138.2(263) | 175.2(±175.6) | 0.0617 |
| ***IFNβ1* ^a^** | 24(43.1) | 93.2(±191.2) | 148.5(220.9) | 156(±137.5) | 0.0334* |
| ***IFNαR2* ^a^** | 75.9(117.5) | 85.9(±55.6) | 41.4(67) | 62.4(±59.5) | 0.2535 |
| ***RNF114* ^b^** | 141.3(63.7) | 134.4(±33.1) | 145.9(87.2) | 147.7(±56.3) | 1.0 |
| ***RNF125* ^b^** | 36.3(27.8) | 40.8(±28.3) | 39.6(27.6) | 38.6(±12.8) | 0.62 |
| ***UCHL1* ^b^** | 11(27.7) | 20(±17.3) | 10.6(13.2) | 14.8(±17.6) | 0.9015 |
| ***TNFα* ^c^** | 4.5(16.6) | 8.8(±8.8) | 6.3(11.3) | 8.2(±6) | 0.7959 |
| ***IFNγ* ^d^** | 19.4(35.2) | 26.8(±24.9) | 56.0(165) | 104.0(±132.6) | 0.1655 |
| ***MCP1* ^e^** | 0(43.9) | 50.3(±11.3) | 0(11.9) | 7.2(±15) | 0.4489 |
| ***MIP-1α* ^e^** | 11(308.3) | 564.9(±1371) | 0(350.4) | 489.9(±1229) | 0.3774 |
| ***MIP-1β* ^e^** | 0(199.2) | 179.7(±334.4) | 0(148.6) | 96.5(±203.4) | 0.4461 |
| ***IP-10* ^e^** | 0(0) | 20.3(±70.3) | 0(2.1) | 3.1(±7.7) | 0.4838 |
| ***IL-8* ^e^** | 11.5(13889) | 11476(±26736) | 0(48698) | 23141(±46041) | 0.3446 |
| ***IL-10* ^e^** | 0(0) | 3.3(±11.5) | 0(0) | 1.3(±3.9) | 0.9444 |
| ***IL-1β* ^e^** | 9.9(3431) | 2021(±3805) | 0(1183) | 2599(±6955) | 0.3497 |
| ***IL-6* ^e^** | 0(0) | 0.6(±1.9) | 0(0) | 1.5(±4.6) | 0.8343 |
| ***E-caderin* ^e^** | 0(187.7) | 139.9(±278.4) | 0(19.43) | 22.5(±54.5) | 0.3096 |
| ***Claudin 1* ^e^** | 0(145.1) | 87.2(±164) | 0(21.4) | 19.1(±43.5) | 0.3096 |
| ***Claudin 2* ^e^** | 0(0) | 0(0) | 0(0) | 0(0) | - |
| ***Claudin 4* ^e^** | 1316(1499) | 1709(±1166) | 1136(714) | 1279(±841) | 0.3374 |
| ***Ocludin* ^e^** | 0(120.4) | 69.7(±114.9) | 0(59) | 302(±862.4) | 0.7759 |
| ***ZO-1* ^e^** | 0(502.8) | 286.6(±419.3) | 0(155.8) | 113.1(±245.1) | 0.3517 |

IQR – interquartile range; a – HPV- participants = 15 and HPV+ participants = 11; b – HPV- participants = 7 and HPV+ participants = 7; c - HPV- participants = 8 and HPV+ participants = 4; d - HPV- participants = 10 and HPV+ participants = 10; e - HPV- participants = 12 and HPV+ participants = 9. * - *p value* ≤ 0.05.
